# Fecal microbiota transplantation for refractory diarrhea in immunocompromised diseases: a pediatric case report

**DOI:** 10.1186/s13052-019-0708-9

**Published:** 2019-08-28

**Authors:** Shuwen Zhong, Jingqing Zeng, Zhaohui Deng, Lirong Jiang, Bin Zhang, Kaihua Yang, Wenyu Wang, Tianao Zhang

**Affiliations:** Department of Pediatric Gastroenterology, Shanghai Children’s Medical Center, School of Medicine, Shanghai Jiao Tong University, No. 1678 Dongfang Road, Pudong Area, Shanghai, 200127 China

**Keywords:** Fecal microbiota transplantation, Refractory diarrhea, Graft-versus-host disease, Immunocompromised children

## Abstract

**Background:**

Immunocompromised (IC) patients have an increased risk of refractory diarrhea. Fecal microbiota transplantation (FMT) is a safe and effective therapy for infection-related diarrhea which are mainly mediated by the loss of the microbial colonization, although there is concern that IC patients may be at higher risk of infectious complications related to FMT. And reports of FMT in IC children are limited.

**Case presentation:**

We describe two cases of FMT in IC children with refractory diarrhea. One IC child had polyendocrinopathy, enteropathy, X-linked syndrome and the other child had graft-versus-host disease. Both of the children had a long course of diarrhea and no response to traditional treatment. FMT was performed on both patients via nasojejunal tubes under guidance of gastroduodenoscopy. After FMT, the patients achieved remission of symptoms and neither of them had related infectious complications. Microbiota analysis showed that FMT resulted in reconstruction of a diverse microbiota.

**Conclusions:**

Use of FMT is safe and effective in treatment of refractory diarrhea in IC children with a damaged microbiota.

## Background

Diarrhea is the most common symptom in immunocompromised (IC) children. Prophylactic, prolonged, and repeated antimicrobial use and an IC status causing intestinal flora disorders can contribute to an increased risk for diarrhea [1, 2]. Microbial dysbiosis leads to endotoxin translocation, aggravates the inflammatory response, and further damages immune function [3–5]. Therefore, restoration of the microbiota to a homeostatic state plays a critical role in preventing progression of diarrhea in an IC status. Fecal microbiota transplantation (FMT) is increasingly being used as an effective therapy for recurrent *Clostridium difficile* infection (CDI) in adults when standard treatments have failed [6–8]. However, the use of FMT among IC patients has been limited because of concerns about its safety in this population. To the best of our knowledge, there are limited data on FMT in children, especially IC children [9]. We report here two consecutive IC children who received FMT at our institution.

## Case presentation

### Case 1

After 1 month of antibiotics for repeated pneumonia, a 2-year-old boy with a history of polyendocrinopathy, enteropathy, X-linked syndrome presented with watery diarrhea (type VII according to the Bristol Stool Scale) for longer than 4 months. Stool culture results were normal. *Clostridium difficile* antigen and the toxin B gene of stool were negative. This patient was treated with smectite powder, racecadotril granules, *Lactobacillus* probiotics, and rehydration. There was no significant improvement in the child, and his weight was reduced from 12 to 8 kg throughout this period. He also suffered from hypokalemia, acidosis, and severe malnutrition. Electrolyte replacement, total parenteral nutrition (TPN), and immunoglobulin were then administered. Because of ongoing diarrhea that was unresponsive to conventional treatment, the patient finally underwent two times of FMT via a jejunal tube under the guidance of gastroduodenoscopy. During a 7-day follow-up after the first FMT, the frequency of bowel movement decreased from 10 times to four times per day and the shape of the stool was obviously improved. TPN was stopped 1 week after FMT. However, on the 9th day after transplantation, urinary tract infection was confirmed by a swollen urethra opening with intermittent pus discharge. The white blood cell count was more than 50 in each high-power field as shown by a routine urine test. Cefuroxime was initially used as an empirical antibiotic, and then piperacillin/ tazobacta and meropenem were administered successively according to urine culture and drug sensitive test result. The stool mass was increased again on the 16th day after the first FMT. A second FMT was performed in the same manner on the 20th day after the first FMT. The FMT procedures were well tolerated with no adverse events, such as vomiting, abdominal distention, and fever. Four weeks after the second FMT, his stool was observed once a day, and the shape of the feces was type III according to the Bristol Stool Scale. His weight increased to 10 kg 1 month after FMT, and it was 11.4 kg in the second month and 12.4 kg in the third month. Allogeneic hematopoietic stem cell transplantation was successfully performed at 3 months after FMT.

### Case 2

A 5-year-old boy was diagnosed with Wiskott–Aldrich syndrome (WAS) in October 2016. He received graft form 9/10 HLA-matched peripheral blood stem cells of his mother on 4 May 2017. He presented with a 2-month history of recurrent diarrhea after hematopoietic stem cell transplantation. Cyclosporin, mycophenolate mofetil, and methotrexate were initially used for graft-versus-host disease (GVHD) prophylaxis. A rash occurred on day + 4 after transplantation and watery stool occurred on day + 6. Smectite powder and racecadotril powder were then applied to reduce the symptoms. Intravenous methylprednisolone (2 mg/kg/d) was administered on day + 10. GVHD grade was evaluated as III and then basiliximab, tacrolimus, and sirolimus were successively used to reduce acute GVHD. However, the patient did not respond to these strategies. The patient developed abdominal pain, abdominal tenderness, and worsened diarrhea when the stool volume reached 1500 mL/d on day + 35, and infliximab was then administered. He had intermittent fever and anti-infective therapy (meropenem, vancomycin, micafungin sodium, amikacin, fluconazole, sulfamethoxazole) was administered. Abdominal ultrasound and an X-ray showed the presence of intestinal obstruction. Multiple fluid levels were observed in the upper abdomen. Non-surgical therapy was then applied, including fasting, gastrointestinal decompression, maintenance of water–electrolyte balance, blood transfusion, TPN, and effective antibiotics. Bloody stool occurred on day + 37. Pelvic computed tomography showed edema and thickening of the intestinal wall, and pelvic intestinal effusion accompanied by some intraluminal high-density lesions. The patient then had surgical consultation. The surgeon suggested continuing the medical treatment without surgery because of intestinal rejection, the wide range of lesions, and the complex condition of the patient. Although the abdominal pain was relieved, the color of the stool turned yellow-green on day + 49, but diarrhea was not alleviated. Stool screening showed no CDI. Because the diarrhea persisted for longer than 2 months with traditional methods to relieve GVHD, we attempted to use FMT to treat diarrhea.

Fecal suspension was administered via a jejunal tube with the help of gastroduodenoscopy on day + 75. Consequently, the procedure of a second FMT was repeated 2 days later. The patient had no adverse reactions. Stool volumes were deceased from 1500 to 200 mL/d after FMT and TPN was stopped on day + 90. The patient’s stool returned to normal on day + 103 and he was maintained on tacrolimus and cyclosporine. The patient did not have diarrhea at 3 months of follow-up.

### Process of FMT and microbiota analysis

#### Donor selection

Donated stool for FMT was obtained from a 6-year-old healthy boy. The donor hadn’t taken antibiotics or probiotics within the past 3 months. The parents of the donor were required to complete a Donor Questionnaire form. To prevent transmission of infectious diseases from the donor to the recipient, the donor underwent stool test screening (microscopy, stool culture, rotavirus antigen, ova and parasite detection, *Clostridium difficile* toxin, *Helicobacter pylori* stool antigen, and Cryptosporidium, Giardia, and Isospora antigens) and serological tests (hepatitis A virus, hepatitis B virus, hepatitis C virus, HIV 1&2 antibody, and syphilis antibody) on the basis of published practice [10, 11].

#### Tests for the recipients

The recipients were tested for hepatitis B antigen, hepatitis C antibody, HIV antibody, and syphilis antibody, and the stool samples were tested for CDI. Antibiotics were stopped the night before FMT. The recipients fasted for at least 6 h before FMT. A detailed explanation of FMT was provided to the recipients. Our study was conducted in accordance with the Declaration of Helsinki. Informed consent was obtained from both of the parents of the stool donor and the FMT recipients. The study was approved by the hospital ethics committee.

#### FMT procedure

Fresh fecal samples from the healthy donor were collected using sterile bags and immediately transported to the laboratory. For each specimen, the stool sample was stored in a liquid nitrogen tank according to the protocol described by published practice [11, 12] with minor modifications. Briefly, approximately 30 g of the stool sample was diluted with 100 mL of sterile saline. A low setting of speed was used until the sample broke up, and then the speed was gradually increased to the highest setting and was continued for 2–4 min until the sample was smooth. The suspension was filtered using a 90-mm perforated filter plate and the sample was collected in a sterile container with a capacity of 100 mL. The sample was used to perform FMT within 6 h.

FMT was performed by an experienced pediatrician via a nasojejunal tube under guidance of gastroduodenoscopy. Anesthesia was monitored during the procedure. An X ray was obtained before transplantation to ensure that the tube was well positioned.

#### Microbiota analysis

Microbial DNA was extracted from samples using the E.Z.N.A.® soil DNA Kit (Omega Bio-tek, Norcross, GA, USA) according to the manufacturer’s protocols. The V3–V4 hypervariable regions of the bacteria 16S rRNA gene were amplified with the primers 338F (5′-ACTCCTACGGGAGGCAGCAG-3′) and 806R (5′-GGACTACHVGGGTWTCTAAT-3′) by a GeneAmp 9700 PCR System (Applied Biosystems, Foster City, CA, USA). PCR reactions were performed in triplicate with a 20-μL mixture containing 4 μL of 5 × FastPfu Buffer, 2 μL of 2.5 mM dNTPs, 0.8 μL of each primer (5 μM), 0.4 μL of FastPfu Polymerase, and 10 ng of template DNA under the following cycling conditions: 3 min of denaturation at 95 °C, 27 cycles of 30 s at 95 °C, 30 s for annealing at 55 °C, and 45 s for elongation at 72 °C, and a final extension at 72 °C for 10 min. The resulting PCR products were extracted from a 2% agarose gel and further purified using the AxyPrep DNA Gel Extraction Kit (Axygen Biosciences, Union City, CA, USA) and quantified using QuantiFluor™-ST (Promega, Madison, WI, USA) according to the manufacturer’s protocol.

Purified amplicons were pooled in equimolar amounts and paired-end sequenced (2 × 300) on an Illumina MiSeq platform (Illumina, San Diego, CA, USA) according to the standard protocols by Majorbio Bio-Pharm Technology Co. Ltd. (Shanghai, China). Raw fastq files were demultiplexed, quality-filtered by Trimmomatic, and merged by FLASH. Operational taxonomic units were clustered with 97% similarity cutoff using UPARSE (version 7.1, http://drive5.com/uparse/), and chimeric sequences were identified and removed using UCHIME. The taxonomy of each 16S rRNA gene sequence was analyzed by the RDP Classifier algorithm (http://rdp.cme.msu.edu/) against the Silva (SSU128) 16S rRNA database using a confidence threshold of 70%.

#### Follow-up and outcomes

**Fig. 1:**
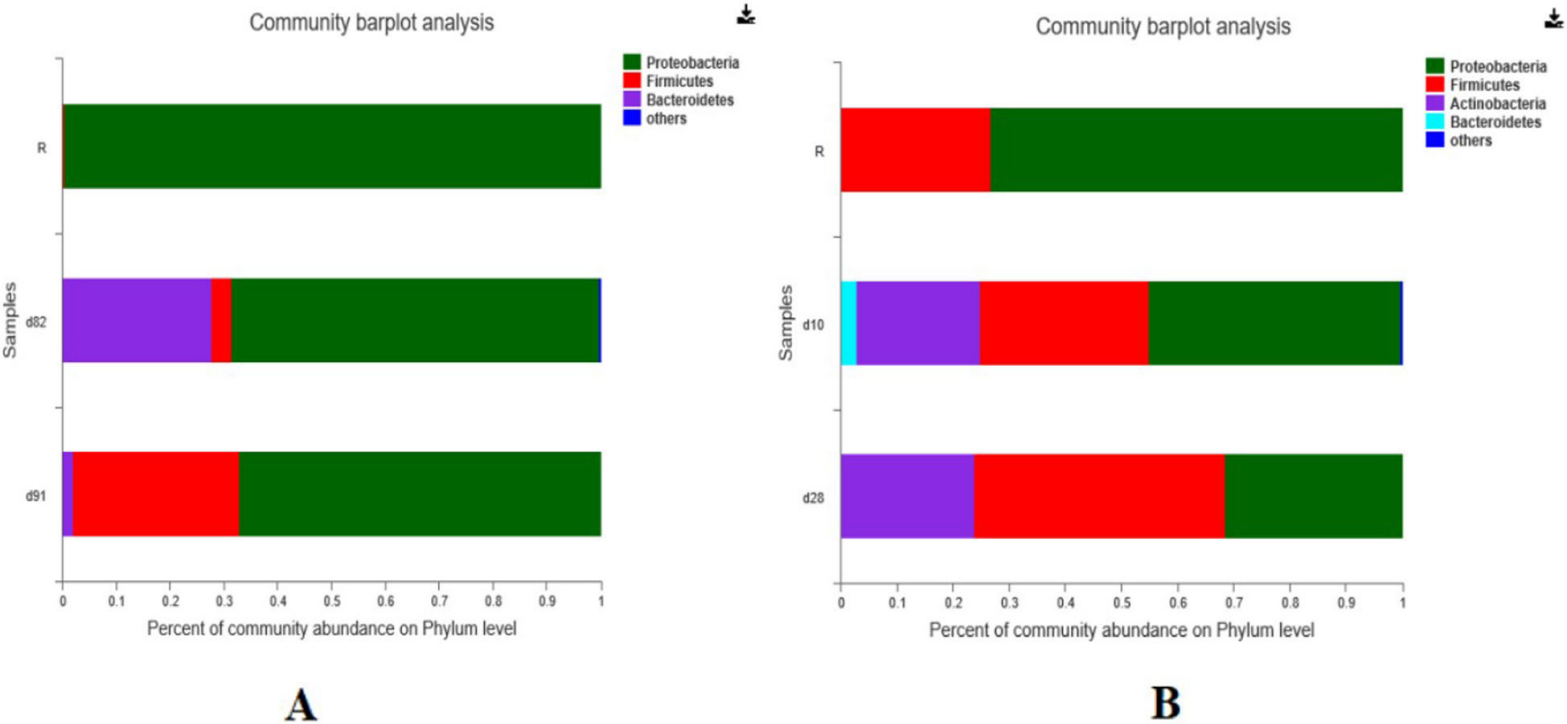
Changes in bacterial community composition after FMT in patient 1(**a**) and patient 2(**b**)

FMT in IC children with refractory diarrhea was successful. Stool samples were collected from recipients before FMT. Additional samples were taken from the recipients at different times after transplantation. The relative abundance of bacteria at the phylum level in two patients’ feces is shown in Fig. 1. Taxonomic analysis showed that the most prevalent phylum was Proteobacteria before FMT. After FMT, Proteobacteria gradually decreased in the patients’ post-FMT samples. In contrast, Firmicutes increased with time.

**Fig. 2:**
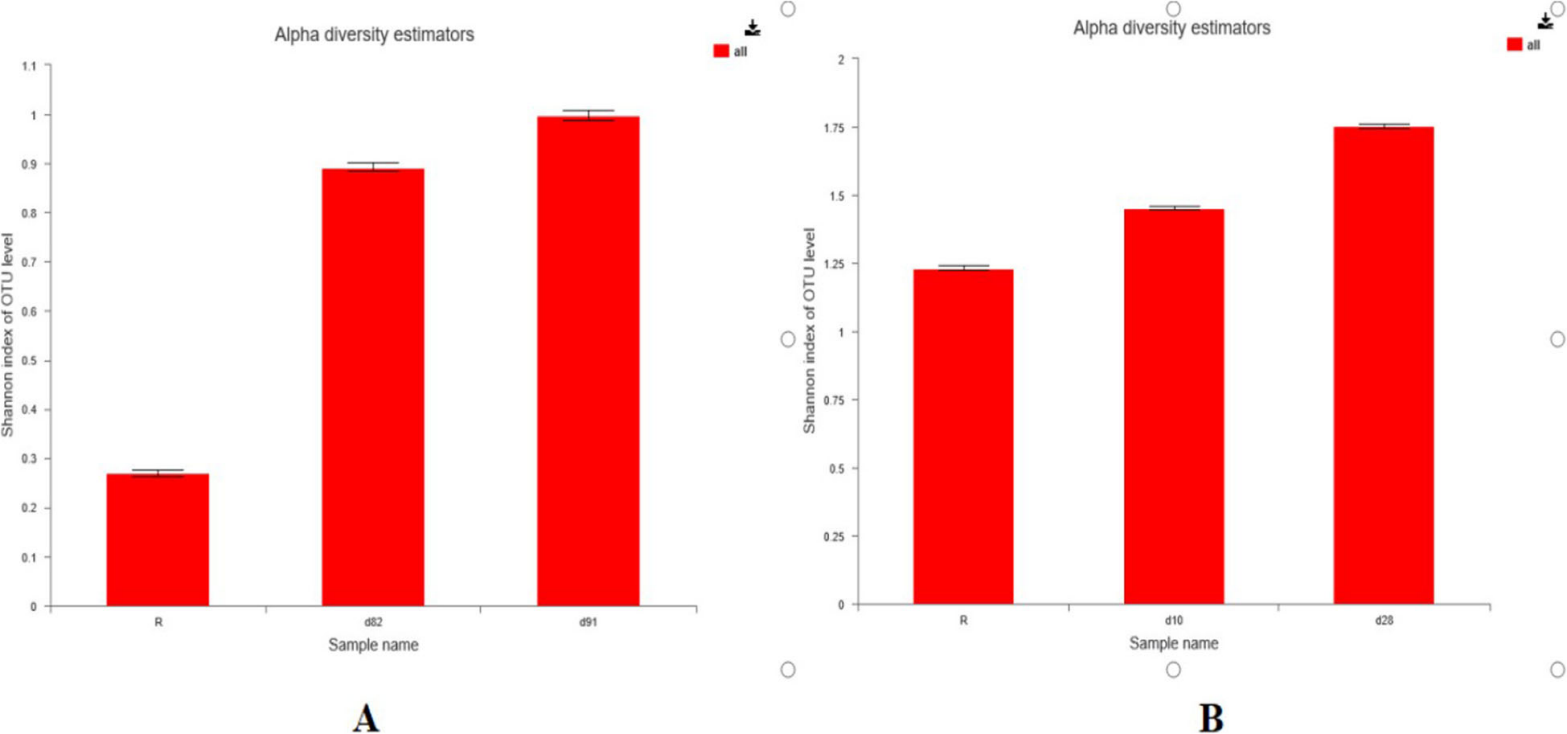
Shannon (alpha) diversity reflects diversity of patient 1(**a**) and patient 2(**b**)

After FMT, the microbial community was more diverse in the two recipients. Analysis of the microbial diversity (Shannon’s index) showed that the index increased rapidly over time (Fig. 2).

## Discussion and conclusions

FMT has been shown to be safe and efficacious in numerous cases and in a recently published clinical trial, especially in treatment of CDI and inflammatory bowel disease [6–8, 13]. However, use of FMT among IC patients has been limited because of concerns about its infectious complications. IC patients include patients treated with immune suppressant medication after solid organ transplantation, decompensated liver cirrhosis, and HIV/AIDS infection [14]. If these patients receive FMT, they have a potential risk for bloodstream infections. Therefore, IC patients were excluded from the first randomized trial of FMT [15]. In our study, the patients had refractory diarrhea because of extensive antibiotic history owing to pneumonia and GVHD following marrow transplantation. They had a long course of diarrhea for several months and considerable weight loss. Traditional treatment was not successful for both of the patients. Fortunately, after FMT, the recipients achieved remission of symptoms and neither of them had related infectious complications.

FMT was successfully applied to treat several recurrent CDIs in patients with solid organ transplantation who did not respond to standard therapy [11, 16, 17]. The safety of FMT was well within the follow-up period and no complications occurred in these studies. Colleen et al. [14] further evaluated the response and serious adverse event rates of FMT for CDI in IC patients. These authors found that the overall cure rate was 89%, which is similar to the 80–90% success rates reported in the general population [15, 18]. Serious adverse events in their study were observed in 12 (15%) IC patients within 12 weeks post-FMT [14]. Two deaths occurred. One patient died 13 days post-FMT with death due to progressive pneumonia, while the second patient died 1 day after FMT following aspiration pneumonitis during sedation for colonoscopy. However, whether those deaths were directly related to FMT, to CD infection, or to the patient’s underlying immunocompromised state was not determined.

Methods for delivery of a liquid suspension of donor stool to the recipient can be classified into upper gastrointestinal routes (nasogastric tube, nasojejunal tube, esophagogastroduodenoscopy) and lower gastrointestinal routes (colonoscopy and enemas) [19]. However, the best route of administering a fecal suspension has not been established. Postigo et al. [19] did not find a significant difference in efficacy between lower gastrointestinal and upper gastrointestinal delivery of FMT (95% vs. 88%) (*P* = 0.162). Gundacker et al. [20] showed that FMT by a nasogastric tube was less effective than that by colonoscopy. However, a small randomized study of 20 patients by Youngster et al. showed that colonoscopy and a nasogastric tube were equally successful [21]. Oral intake of microbiota capsules is the latest mode of stool delivery in FMT [22]. Capsules are a relatively noninvasive, convenient, and safe procedure for eliminating the risk of perforation by endoscopy. Unfortunately, capsules were not available in our hospital when our patients were being treated. Both patients had FMT performed via nasojejunal tubes under guidance of gastroduodenoscopy. In case 1, we chose upper gastrointestinal delivery because this was the choice of the parents. Colonoscopy was refused by his parents because of his young age. Upper gastrointestinal routes are typically faster, less expensive, and better tolerated compared with colonoscopy, although they are not as esthetically pleasing to some patients [23]. In case 2, we chose upper gastrointestinal delivery because of the high risk of intestinal perforation by colonoscopy. Colonoscopic FMT has the advantage of the capacity to deliver fecal infusion directly to the colon. However, the patient was severely ill and he could not tolerate the colonoscopic procedures. Abdominal tenderness was observed by a clinician and considerable colonic distention was confirmed by an imaging examination. Under these circumstances, we preferred the safer and faster method of FMT for this patient.

Recent awareness of the importance of the gut microbiome in human health has greatly improved our understanding of the interactions between gastrointestinal bacteria and the immune system. Furthermore, maintaining healthy microbial communities at mucosal surfaces is important. In IC patients, changes in the gut microbiome could contribute to an increased risk for diarrhea. The delicate balance of commensal bacterial communities is perturbed, resulting in microbial dysbiosis, and an altered gut microbiome is associated with mucosal dysfunction, systemic inflflammation, and disease progression. FMT is a promising therapeutic option that can balance the microbiome community. In our patients, microbiota analysis by 16S rRNA gene sequencing before FMT showed a severely depleted microbiota in both patients, characterized by increased Proteobacteria and decreased Firmicutes. After FMT, the patients’ microbiota was no longer dominated by Proteobacteria and Firmicutes, and the diversity of the gut microbiota increased.

Our observed change in the gut bacterial microbiome is similar to that after FMT in pediatric heart transplantation [17]. Stripling et al. [11] found that a single heart–kidney transplant recipient with recurrent *Clostridium difficile* had a gut microbiota dominated by Enterococcus (relative abundance of 84%) before FMT. There was also a remarkable decline in the relative fecal abundance of Enterococcus after FMT with increased sample diversity.

In conclusion, FMT is safe and effective for treating refractory diarrhea in IC children with a damaged microbiota. FMT results in reconstruction of a diverse microbiota. There are some limitations of our study. Despite the initial successful use of FMT in our IC children, the specific mechanisms of FMT, selection of the donor, and the optimal dose and timing required for a successful transplant still need to be clarified. Therefore, use of FMT requires high-quality, prospective, randomized, controlled trials with a large sample in the field of pediatric diseases.

## Data Availability

The datasets used or analysed during the current study are available from the corresponding author on reasonable request.

## Abbreviations

CDI: *Clostridium difficile* infection
FMT: fecal microbiota transplantation
GVHD: graft-versus-host disease
IC: immunocompromised
TPN: total parenteral nutrition
